# Supplementary material for: Limitations of the radiotheranostic concept in neuroendocrine tumors due to lineage-dependent somatostatin receptor expression on hematopoietic stem and progenitor cells
Source: Theranostics. 2025 May 25;15(13):6497–515. doi: 10.7150/thno.113354 (PMC12160025; doi:10.7150/thno.113354)
Supplement: Supplementary file 1 — Supplementary figures and tables. [file thnov15p6497s1.pdf]

Supplementary Figures:

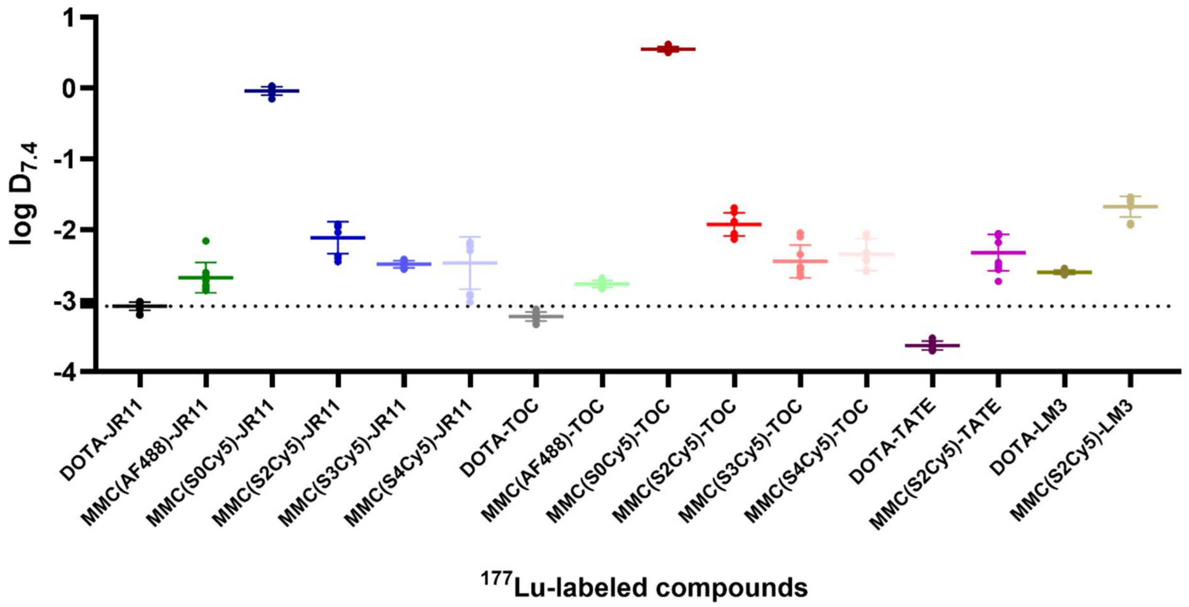

Supplementary Figure 1: Determination of the log $D_{7.4}$ value (lower values = more hydrophilic) of the MMC(Dye)-ligands TOC, TATE, JR11 and LM3 and their respective DOTA-peptide.

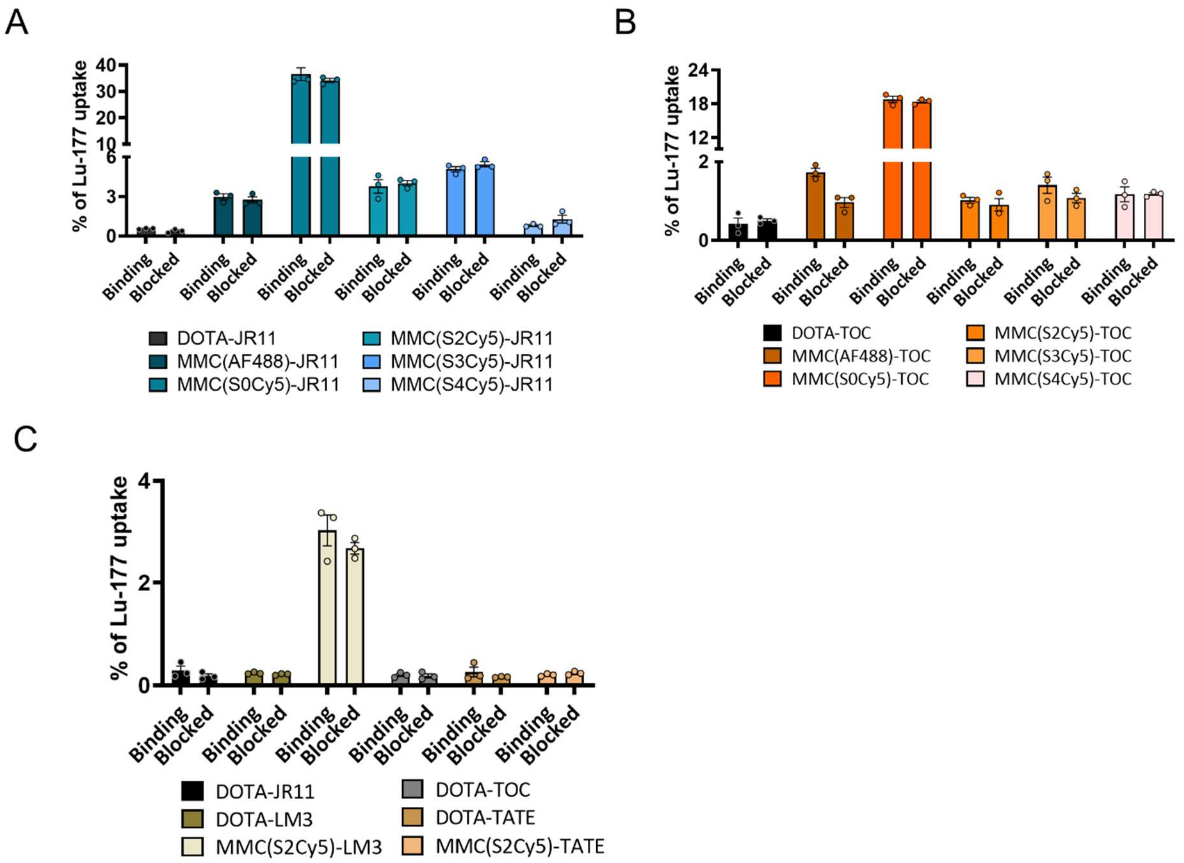

Supplementary Figure 2: Radioligand characterization on HCT116-WT cells. Cellular uptake of ^{177}Lu-labeled JR11 **(A)**, TOC **(B),** LM3 and TATE **(C)** analogs, reported as % uptake of total added ^{177}Lu-activity on SSTR2-negative HCT116-WT cells with or without blocking with a 1000-fold excess of DOTA-JR11.

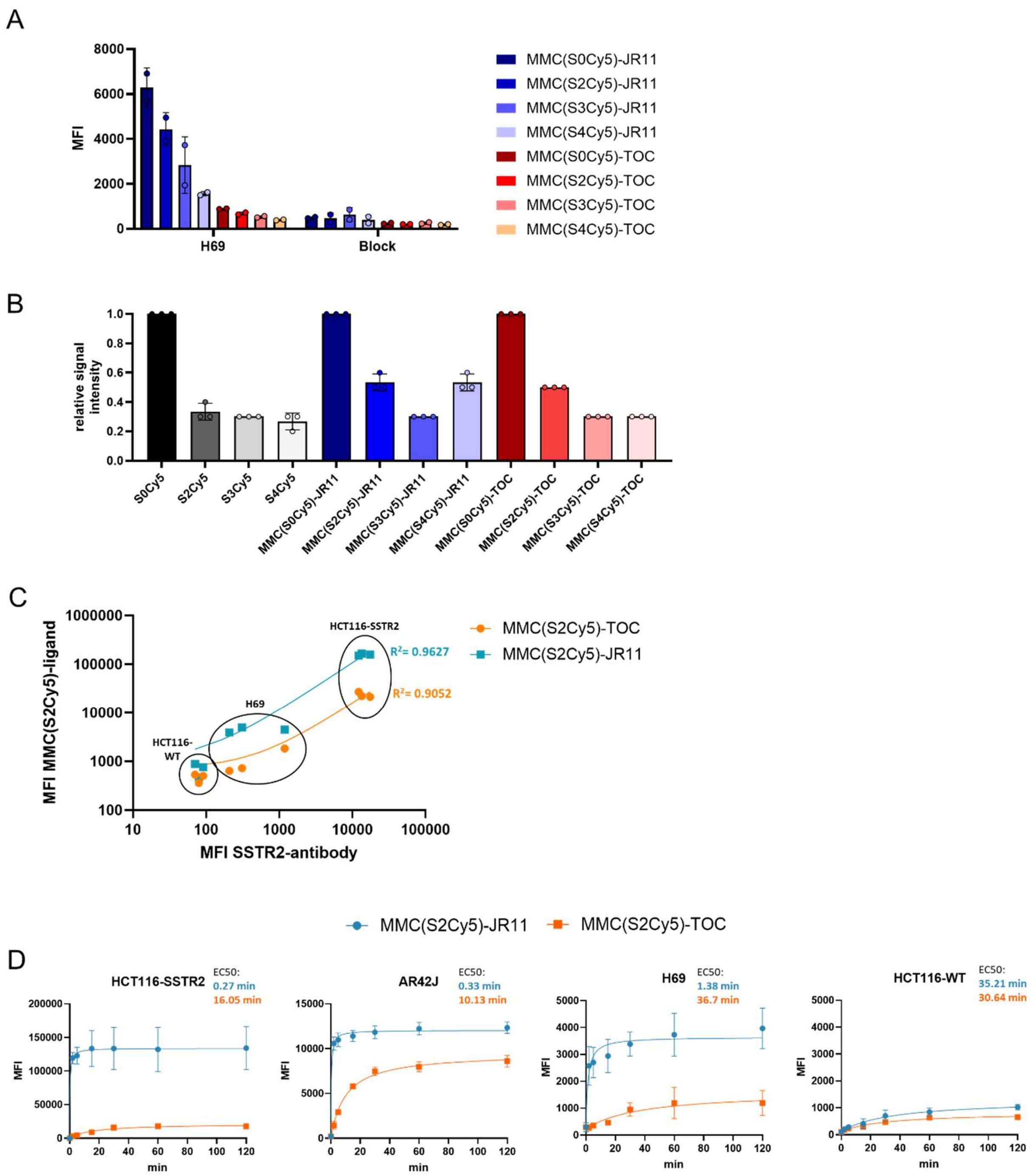

Supplementary Figure 3: **A)** Cellular uptake of MMC(Dye)-ligand reported as mean fluorescence intensity (MFI) on AR42J and H69 cells with or without blocking with 100-fold excess DOTA-JR11. **B)** Fluorescence intensity comparison of MMC(Dye)-conjugates and unconjugated DBCO-dyes in 5 µM DMSO. Intensities were normalized to the brightest dye (S0Cy5-DBCO). **C)** SSTR2 antibody binding in correlation to multimodal ligand binding in tumor cells. R^2 values were derived from linear regression. **D)** Time-dependent cellular uptake of MMC(S2Cy5)-ligands reported as mean fluorescence intensity (MFI) for HCT116-SSTR2, AR42-J, H69 and negative control cell line HCT116-WT. JR11-based compounds show higher MFI and faster cell association in SSTR2-expressing cell lines as determined by EC_{50} values.

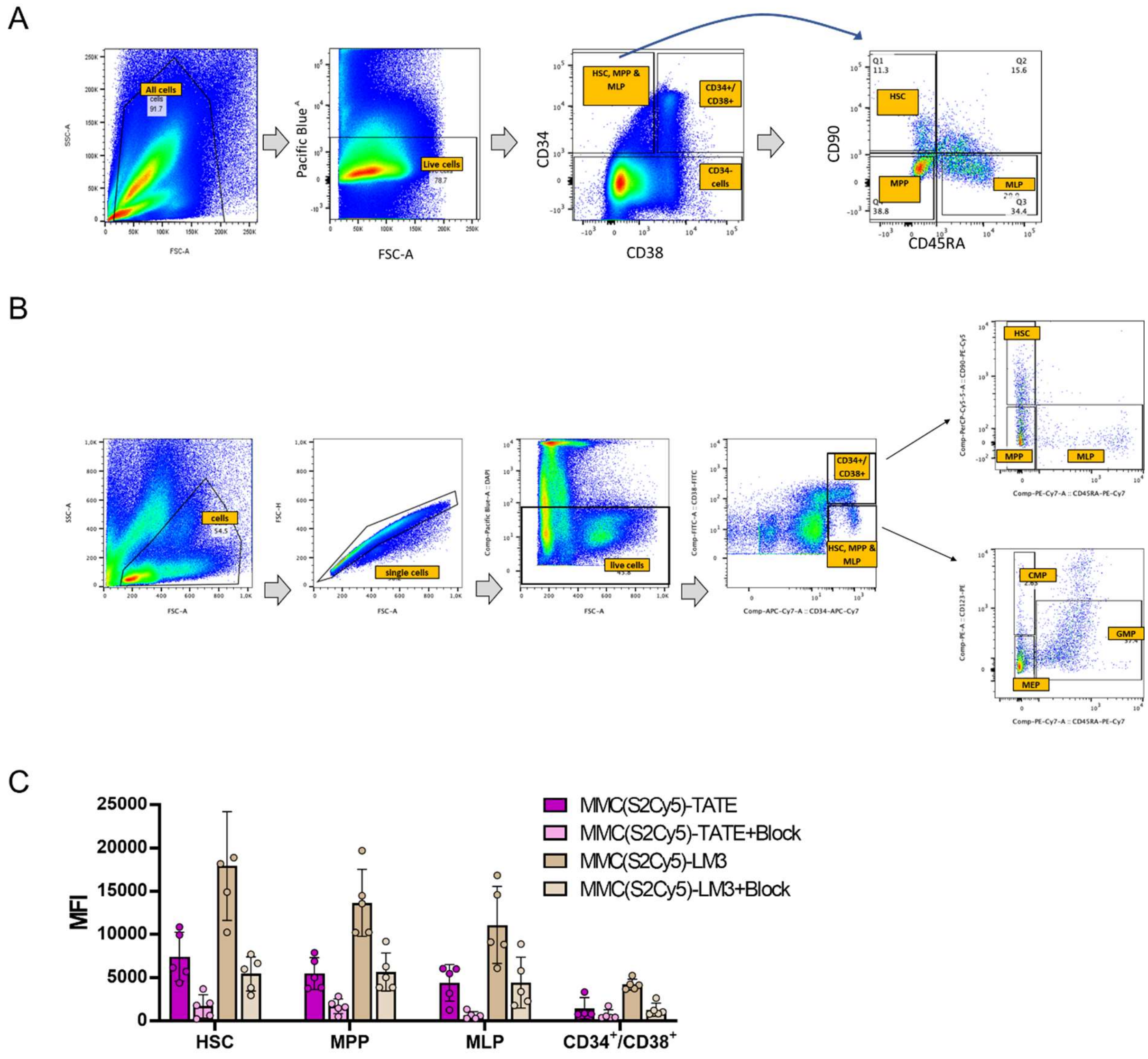

Supplementary Figure 4: A) Multicolor flow cytometry gating strategy were employed for the identification of the 4 human HSC subpopulations HSC, MPP, MLP and CD34+/CD38+ based on CD-markers CD34, CD38, CD90 and CD45RA. **B)** Multicolor flow cytometry gating strategy for the identification of the 6 human HSC subpopulations HSC, MPP, MLP and CMP, MEP and GMP (together they represent CD34+/CD38+) based on CD-markers CD34, CD38, CD90, CD45RA and CD123. **C)** MFI of MMC(S2Cy5)-TATE/LM3 in HSPC subpopulations with or without blocking.

Supplementary Table:

Supplementary Table 1: Age and gender distribution of the treatment cohort. Age in years (y) is given as mean and (range).

	Total	Female	Male
Number (n)	16	8	8
Age (y)	52.875 (32-59)	50.375 (32-59)	55.375 (46-58)
High Sensitivity (n)	8	4	4
Low Sensitivity cohort (n)	8	4	4
High Sensitivity (y)	50.25	47.0 (32-55)	53.5 (46-58)
Low Sensitivity cohort (y)	55.5	53.75 (46-58)	57.25 (55-58)
